# Raise Your Standard of Efficiency

**Published:** 1914-01-24

**Authors:** 


### Raise Your Standard of Efficiency.

### WEST NORFOLK AND LYNN HOSPITAL.

Sir Somerville Gurney, the chairman of the finance committee of the West Norfolk and Lynn Hospital, has written a letter to the *Eastern Daily Press* and to several of the Lynn papers agreeing with some and disagreeing with other portions of Sir Henry Burdett's Report on this hospital published in The Hospital of the 10th inst., page 397. We could wish that the Lynn papers had followed the universal practice and published Sir Henry's Report in full, so that the people of Lynn might have had a full opportunity of judging that Report as a whole and forming their own conclusions upon it.

Sir Henry Burdett's statements as to the former condition of the hospital, and the possibility, by a supreme effort, to have raised a building fund of sufficient amount to have enabled the hospital to be rebuilt a few years ago, were based upon internal evidence and authoritative statements made to him as the result of his inquiries. Sir Somerville Gurney has no doubt grown up with the hospital as it is, and to him, as to many others, familiarity tends to soften abuses and may even destroy the power to observe closely and to realise actual conditions as they in fact are, when judged from the standpoint of a modern hospital. Whilst we welcome Sir Somerville Gurney's letter as evidence of his desire to co-operate in making the West Norfolk and Lynn Hospital thoroughly efficient, we would beg of him to visit the Norfolk and Norwich Hospital and the Cromer Hospital, just to see how different a hospital ward becomes when the comfort of the patients is fully considered and provided for. Sir Henry found in the wards of the West Norfolk and Lynn Hospital practically no furniture or comforts except the beds and bedsteads. He found no day rooms, but simply two passage-ways which were called day rooms; and the furniture in the men's passage-way was old, decrepit, and had the worst features of the old-fashioned, out-of-date workhouse type. Sir Somerville Gurney describes the wards as cheerful and sunny and the passageways as satisfactory. Thus the different points of view taken by each observer are:—

(1) Sir Henry's inspections have been made in the hope of raising the equipment and administration of the principal hospitals throughout England to the standard of an up-to-date modern hospital.

(2) The chairman of the finance committee of the Lynn Hospital apparently regards the defects in his hospital as satisfactory and good enough for the purpose.

We hope Sir Somerville Gurney, the inhabitants of Lynn, and the subscribers to the West Norfolk and Lynn Hospital will make it their business to inspect the two other Norfolk hospitals we have mentioned, and compare them with their own. Then we are confident the necessary funds will be forthcoming, and Lynn will get without unnecessary delay an adequately equipped and more up-to-date and comfortable hospital for the patients, than it possesses at the moment.

